# Factors that affect career success of nurses who practice in assisted reproductive technology *

**DOI:** 10.1590/1518-8345.6388.3927

**Published:** 2023-05-12

**Authors:** Wang Li, Wen Honggui, Peng Hong, Luo Hong

**Affiliations:** 1 Women and Children’s Hospital of Chongqing Medical University, Center for Reproductive Medicine, Chongqing, Chongqing, China; 2 Chongqing Health Center for Women and Children, Center for Reproductive Medicine, Chongqing, Chongqing, China.

**Keywords:** Career Success, Work Environment, Assisted Reproductive Technology, Nurses, Cross-Sectional Studies, China, Éxito en la Carrera, Lugar de Trabajo, Técnicas Reproductivas Asistidas, Enfermeras y Enfermeros, Estudios Transversales, China, Sucesso Profissional, Local de Trabalho, Tecnologia de Reprodução Assistida, Enfermeiras e Enfermeiros, Estudos Transversais, China

## Abstract

**Objective:**

to examine the relationship between career success and work environment of nurses who practice in assisted reproductive technology and to identify factors that affect career success.

**Method:**

a cross-sectional study conducted in 53 fertility centres in 26 provinces in mainland China. Data were collected using a demographic data questionnaire, a specialised nursing competence questionnaire, the Career-Success Scale, and the Nursing Work Environment Scale. Descriptive and inferential statistics were applied.

**Results:**

597 assisted reproductive technology nurses participated in our survey, and 555 valid questionnaires were collected. Theoverall mean scores for career success and work environment were 3.75 [standard deviation (SD) = 1.01] and 3.42 (SD = 0.77) respectively. There was a strong positive correlation between career success and work environment (r = 0.742, p < 0.01). Multiple regression showed that attending academic conferences, psychological care, professional development, support and care, salary, and welfare were significant factors that influence career success.

**Conclusion:**

attending academic conferences, psychological care, and work environment are positively related to career success. Administrators should consider ways to address these factors.

## Destacados

**(1)** Estudio multicéntrico en 53 centros de fertilidad para el éxito en la carrera de las enfermeras.

**(2)** Marcada correlación positiva entre éxito en la carrera y ambiente de trabajo.

**(3)** La concurrencia a conferencias académicas estuvo positivamente relacionada al éxito en la carrera.

**(4)** La atención psicológica estuvo positivamente asociada al éxito en la carrera.

**(5)** Enfocarse en el ambiente de trabajo de enfermería para mejorar el éxito en la carrera de las enfermeras.

## Abstract (pt)

**Objetivo:**

examinar a relação entre o sucesso profissional e o ambiente de trabalho de enfermeiros da área de tecnologia de reprodução assistida e identificar os fatores que afetaram tal sucesso.

**Método:**

estudo transversal conduzido em 53 centros de fertilidade em 26 províncias da China Continental. Os dados foram coletados por meio de um questionário demográfico, um questionário sobre competências de enfermagem especializada em tecnologias de reprodução assistida, da Career-Success Scale (Escala de Sucesso Profissional) e da Nursing Work Environment Scale (Escala do Ambiente de Trabalho na Enfermagem). Aplicaram-se análises de estatística descritiva e inferencial.

**Resultados:**

participaram do estudo 597 enfermeiros da área de tecnologia de reprodução assistida, e 555 questionários válidos foram coletados. As pontuações globais médias para o sucesso profissional e o ambiente de trabalho foram 3,75 [desvio padrão (DP) = 1,01] e 3,42 (DP = 0,77), respectivamente. Verificou-se forte correlação positiva entre o sucesso profissional e o ambiente de trabalho (r = 0,742; p < 0,01). A análise de regressão múltipla mostrou que a participação em eventos acadêmicos, a assistência psicológica, o desenvolvimento profissional, o apoio e os cuidados, e o salário e o bem-estar foram fatores que influenciaram significativamente o sucesso profissional.

**Conclusão:**

a participação em eventos acadêmicos, a assistência psicológica e o ambiente de trabalho mostraram correlação positiva com o sucesso profissional. Os gestores deveriam considerar formas de abordar esses fatores.

**Highlights:**

**(1)** Multicentre study in 53 fertility centres for nurses’ career success.

**(2)** Strong positive correlation between career success and work environment.

**(3)** Attending academic conferences was positively related to career success.

**(4)** Psychological care was positively associated with career success.

**(5)** Focus on nursing work environment to enhance the nurses’ career success.

## Introduction

Infertility is newly defined as “a disease characterized by the failure to establish a clinical pregnancy after 12 months of regular, unprotected sexual intercourse or due to an impairment of a person’s capacity to reproduce either as an individual or with his/her partner” ^( 1)^ . As the infertility incidence is increasing due to greater age and lifestyle and environmental factors ^( 2)^ , so is the number of individuals seeking infertility treatment from healthcare professionals. Assisted reproductive technology (ART) began in the United Kingdom in 1978 with the birth of Louise Brown, the first *in vitro* fertilization (IVF) baby. Since then, nurses have been involved in the treatment, and they play an important role in the management of treatment cycles ^( 3)^ . Research evidence has shown that infertility nursing care can enhance patients’ well-being, treatment compliance ^( 4)^ and quality of life ^( 5)^ . 

However, the increasing number of infertility patients and special occupational characteristics, such as strict protection of patient privacy and being highly involved in the diagnosis and treatment details, can make ART nursing a busy and stressful occupation ^( 6)^ . Meanwhile, compared with rapidly developing reproductive medicine, ART nursing is still a young subject. A recent study discovered a scarcity of assistance models and nursing specialists in the area of ART nursing ^( 7)^ . Currently, infertility nursing undergraduate-level curricula have neither been defined nor established in the university setting, and there has been a limited number of publications related to ART nurses’ career development in mainland China. 

Career success includes positive emotions and achievements pertaining to work that are accumulated and gradually obtained during the work experience ^( 8)^ . At present, the majority of research on career success is concentrated in the field of enterprise management, but research is gradually shifting to the field of nursing ^( 9)^ . Previous studies have shown that career success is helpful to promote innovative behaviour among nurses, and it can improve the quality of nursing service, reduce the turnover rate of nurses, and stabilize nursing teams ^( 10 - 11)^ . Recent studies have shown that demographic characteristics, self-efficacy, social support, information literacy, working in shifts, and having a good individual income and work environment are the influencing factors in nurses’ career success^( 9),( 12) ^. Work environment refers to the organizational characteristics of work settings that facilitate or constrain professional nursing practice. Improving the nursing work environment has been suggested as an effective strategy for addressing the nursing shortage by increasing nurse job satisfaction ^( 10);( 11);( 12);( 13 - 14)^ . Previous research has revealed that the work environment has a significant relationship with career success among nurses ^( 10)^ . 

Thus, understanding how nurses perceive the work environment is important for managers to create a good organisational environment that attracts and retains qualified nurses. In past research, little attention has been paid to examining ART nurses’ careers in China, and there has been a limited number of publications internationally. Research into career success can expand understanding and contribute to educational, governmental, and organisational initiatives that help nurses to achieve career success. Therefore, we conducted a nationwide survey of ART nurses in mainland China to explore the relationship between career success and work environment and the factors that influence career success, in the hope of providing suggestions for promoting ART nurses’ professional well-being and enhancing the quality of the nursing care environment for infertility patients.

## Method

### Study design

A descriptive multicentre study was conducted in accordance with the Strengthening the Reporting of Observational Studies in Epidemiology guidelines (STROBE) for cross-sectional studies. The purpose of the study was to explore the career success and work environment of ART nurses from March to May 2019 in mainland China.

### Sample and setting

Convenience sampling was used to recruit participants. Mainland China is divided into seven geographical regions: East China, South China, Central China, North China, Northwest China, Southwest China, and Northeast China. Three or four provinces were selected from each region using convenience sampling. The fertility centres were categorized according to the hospital type, as follows: teaching hospitals, general hospitals, women and children’s hospitals, traditional Chinese medicine hospitals, and private hospitals. One or three fertility centres were selected from each type using convenience sampling. Finally, 53 fertility centres in 26 provinces in China participated in our study. The sample size was calculated using the G*Power 3.1.2 statistical power analysis software program (Universitat Dusseldorf, Dusseldorf, North-Rhine-Westphalia, Germany). G*Power suggested that we would need 280 participants, with a medium effect size of 0.15, α of 0.05, power of 0.95, and variables of 36. Considering a loss rate of up to 15%, 322 participants would be needed. Five hundred and ninety-seven ART nurses participated in our anonymous online survey. Finally, 555 valid surveys were collected, with an effective recovery rate of 92.96%.

WeChat (a social media application) was used to recruit and sample ART nurses. The ART nurse managers of each infertility setting were interviewed through WeChat and then asked to provide other target subjects in their infertility settings that belonged to the overall research target group. A web version of Questionnaires was used through the professional platform named Wenjuanxin. A web link and quick response (QR) code were generated; participants could access the web version of the questionnaires via the web link or by scanning the QR code through WeChat. All items were mandatory to prevent missing data, and each WeChat account could submit only one of each questionnaire in order to avoid the possibility of double registration. All data were examined for authenticity and credibility, and cases were deleted manually if they met the following criteria: an abnormal questionnaire with obvious regularity or logical confusion.

As for consent, the first page of the questionnaire provided a purpose statement of for this study. The respondents could access the survey questions after they had read the informed consent and agreed to it. The participants were considered to have given informed consent once they had completed and submitted the survey.

The participants were included if they met the following criteria: being a registered nurse; having at least one year of work experience in a fertility centre; and having voluntarily expressed the intention to participate. Nurses on sickness or maternity leave for more than six months were excluded.

### Measures

#### The demographic data questionnaire

Previous literature ^( 10 - 11)^ was used to identify potential variables that may affect nurses’ career success. The questionnaire included seven sociodemographic characteristics (gender, age, marital status, the presence/absence of children, education level, hospital level, and average monthly income), six occupational characteristics (professional title, employment type, work departments before the reproductive department, night shift, years of working experience, and satisfaction with income) and eight research work characteristics (presided over or joined in scientific research projects, academic posts, published papers, been a peer reviewer, undertaken teaching work, participated in academic conferences, undergone further education abroad, and participated in compiling a textbook). Before the formal survey, we invited eight nurse managers in different infertility settings to assess the self-designed questionnaire. As a result, a few alterations were made to the questionnaire design. First, we changed “years of working experience” to “years of working experience in the fertility nursing profession”. Second, we deleted the “employment type” item of occupational characteristics. The content validity index was 0.815. 

#### The ART Specialised Nursing Competence Questionnaire

We developed the ART Specialised Nursing Competence Questionnaire based on literature reviews ^( 15)^ and expert consultation. Considering that our study translated and adapted the English-language questionnaire into the Chinese language, the cultural differences between western countries and China may limit the ability to measure specialized nursing competence in Chinese cultural contexts. The Chinese version of the ART Specialized Nursing Competence Questionnaire was finalized after being well-tested in a representative pilot group of 38 ART nurses. The items included ART specialised knowledge and skills, ART-derived knowledge and skills, health education, counselling skills, psychological care, and ultrasound technique. The score on questionnaire ranged from 1 to 5, where 1 indicated a lack of ART specialised nursing competence and 5 full ART specialised nursing competence. Each item was asked in reference to self-assessment of ART specialised nursing competence and the need for further education. The content validity index for the scale was 0.834, and the Cronbach’s alpha was 0.949. 

#### Chinese version of the Career-Success Scale

The Chinese version of the Career-Success Scale (CSS) is widely used ^( 8)^ . It contains 11 items that are categorized into three subscales: career satisfaction (five items), perceived within-organisation competitiveness (PWOC, three items), and perceived external organisation competitiveness (PEOC, three items). A 5-point Likert-type scale ranging from 1 (strongly disagree) to 5 (strongly agree) is used. The total score ranges from 11 to 55, with higher scores representing higher career success. The Cronbach’s alpha for each subscale was .86 to .87 and .87 for the total scale. Test-retest reliability was 0.93. 

#### The Nursing Work Environment Scale

We measured the nursing work environment using the following subscales of the Chinese version of the Nursing Work Environment Scale ^( 16)^ : professional development (five items), support and care (four items), nurse-physician relationship (four items), recognition of value (three items), clinical autonomy (four items), salary and welfare (three items), and staffing adequacy (three items). This 26-item scale uses a 6-point Likert-type scale (1 = strongly disagree to 6 = strongly agree). The total score ranges from 26 to 156, and higher scores indicate a better work environment, as expressed by the nurses. This scale is a valid and reliable tool, and the content validity index for the total scale was 0.93; the Cronbach’s alpha was 0.94. 

### Data analysis

The data analysis was conducted using SPSS version 22.0 (IBM Corp., Armonk, NY, USA). First, descriptive statistics were used to describe the demographic data questionnaire. Second, the means ( *M*) and standard deviations ( *SD*) were calculated for the ART nurses’ specialised nursing competence, career success, and work environment. Third, *t*-tests and one-way analysis of variance were used to examine the relationship between the demographic characteristics and career success. Fourth, Pearson’s correlations were computed to examine the associations between work environment, ART specialized nursing competence, and career success. Finally, multiple regression analysis was performed to determine the variables that best predict career success. The variance analyses were bilateral, and the significance level was set at *p* < 0.05. 

### Ethical considerations

The study was approved by the Ethics Committee of the Chongqing Reproductive and Genetics Institute (No.2019/608).

## Results

Table 1 shows that the respondents were predominantly female (96.8%), with an average age of 31.3 years. More than 68.0% were married, and 58.4% had children. More than 70.0% had a bachelor’s degree and worked in a tertiary hospital. Most (41.8%) had an average monthly income of 2.5-4 minimum wages (one minimum wage is approximately USD 300) and 40.2% were satisfied with their income. Of the respondents, 75.5% held a junior professional title and nearly two-thirds had work experience before working in infertility settings (64.1%). The average number of working years in infertility settings was 6.4 years. Of the participants, 76.8% had no night shifts and 71.7% were satisfied with their job. Only 7.9% had presided over or joined in scientific research projects and 4.1% had an academic post. Less than 1% were peer reviewers. One-third reported undertaking teaching work, 39.1% had the opportunity to participate in academic conferences, and 18.6% had published a paper. Furthermore, 3.4% and 3.2% had undertaken further education abroad or had experience of textbook compilation, respectively. 

**Table 1 -:** Demographic characteristics and research work characteristics of assisted reproductive technology nurses and their associations with career success (n= 555). Mainland China, 2019

**Characteristics**	**n**	**%**	**Mean**	SD *	***t/F***	***p***
**Gender**						
Male	18	3.2	34.78	7.841	-2.020	0.044
Female	537	96.8	37.70	5.973		
**Age (years)**						
25 and below	99	17.8	36.65	6.591	2.123	0.077
26-30	204	36.8	37.30	6.130		
31-40	196	35.3	37.92	6.014		
41-50	50	9.0	39.50	4.496		
Above 50	6	1.1	37.50	3.987		
**Marital status**						
Not married	176	29.7	36.62	6.326	-2.628	0.009
Married	379	68.3	38.06	5.878		
**Presence or absence of children**						
Absence	231	41.6	36.78	6.357	-2.715	0.007
Presence	324	58.4	38.19	5.770		
**Educational level**						
Diploma	13	2.3	37.16	5.036	2.634	0.049
Associate degree	128	23.1	37.60	5.756		
Bachelor’s degree	406	73.2	38.77	6.901		
Master’s degree	8	1.4	43.13	5.540		
**Hospital level**						
Tertiary hospital	424	76.4	37.93	6.092	1.786	0.149
Secondary hospital	18	3.2	36.72	5.454		
Private hospital	78	14.1	36.35	6.295		
Others	35	6.3	36.97	5.044		
**Average monthly income (MW** ^†^ **)**						
1 to 2.5	92	16.6	36.65	6.554	5.076	0.002
2.5 to 4	232	41.8	36.91	5.962		
4 to 5	115	20.7	38.07	5.775		
Above 5	116	20.9	39.28	5.783		
**Professional title**						
Junior	419	75.5	37.32	6.180	2.374	0.094
Middle	116	20.9	38.29	5.756		
Senior	20	3.6	39.65	4.392		
**Work departments before Reproductive Department**						
There was none	199	35.9	37.39	6.176	0.848	0.548
Surgery department	71	12.8	37.77	6.102		
Medical department	56	10.1	36.88	5.844		
Gynaecology and obstetrics department	162	29.2	38.36	5.827		
Paediatrics department	19	3.4	36.68	7.040		
Operating room	13	2.3	35.77	6.418		
Emergency department	12	2.2	38.42	7.573		
Others	23	4.1	36.78	5.108		
**Night shift**						
Yes	129	23.2	37.09	6.180	-1.114	0.266
No	426	76.8	37.76	6.016		
**Years of working experience in fertility nursing profession**						
4 years and below	236	42.5	37.47	6.360	0.513	0.599
5-9 years	196	35.3	37.94	5.519		
10-20 years	123	22.2	38.34	6.294		
**Job satisfaction**						
Satisfied	398	71.7	38.86	5.564	8.257	0.000
Other (fair/dissatisfied)	157	28.3	34.41	6.095		
**Income Satisfaction**						
Satisfied	223	40.2	39.93	5.331	7.812	0.000
Other (fair/dissatisfied)	332	59.8	36.04	6.018		
**Presiding over or joining in scientific research projects**						
Yes	44	7.9	40.75	4.975	-3.629	0.000
No	511	92.1	37.33	6.068		
**Academic post**						
Yes	23.	4.1	37.47	6.036	-2.512	0.012
No	532	95.9	40.70	5.787		
**Published paper**						
Yes	103	18.6	39.27	5.092	-3.119	0.002
No	452	81.4	37.23	6.197		
**Peer reviewer**						
Yes	4	0.7	36.25	7.890	0.449	0.654
No	551	99.3	37.62	6.048		
**Undertake teaching work**						
Yes	167	30.1	38.62	5.690	-2.610	0.009
No	388	69.9	37.17	6.162		
**Participation in academic conferences**						
Yes	217	39.1	39.19	5.491	-5.059	0.000
No	338	60.9	36.59	6.188		
**Further education abroad**						
Yes	19	3.4	39.21	5.893	-1.176	0.240
No	536	96.6	37.55	6.059		
**Participation in textbook compilation**						
Yes	18	3.2	40.28	3.968	-1.908	0.057
No	537	96.8	37.52	6.095		

* SD = Standard Deviation

† MW = Chinese minimum wage, approximately USD 300 dollars (2,000 Renminbi in Chinese currency)

Table 2 presents the ART nurses’ level of specialised nursing competence. The overall mean score for work environment was 21.92 (SD= 3.540). After calculating the average score of each variable, the results were arranged in descending order as follows: health education, counselling skills, ART specialised knowledge and skills, psychological care, ART-derived knowledge and skills, and ultrasound technique. 

**Table 2 -:** Level of specialized nursing competence in assisted reproductive technology nurses (n=555). Mainland China, 2019

**Variables**	Mean ± SD *
Health education	3.88±0.667
Counselling skills	3.79±0.675
ART specialized knowledge and skills	3.75±0.716
Psychological care	3.63±0.735
ART-Derived knowledge and skills	3.53±0.803
Ultrasound technique	3.25±0.929

* SD = Standard deviation

The ART nurses’ work environment and career success scores are shown in Table 3. The overall mean score for work environment was 3.75 (SD= 1.010) and the recognition of value dimension (M= 4.13, SD= 0.923) had the highest mean. The staffing adequacy dimension (M= 3.40, SD= 1.016) had the lowest mean. The overall mean score for career success was 3.42 (SD= 0.765), and among the three dimensions, the career satisfaction score was highest (M= 3.58, SD= 0.721), while the PEOC was lowest (M= 3.21, SD = 0.751). 

**Table 3 -:** Score for work environment and career success in assisted reproductive technology nurses (n= 555). Mainland China, 2019

**Variables**	**Items**	**Range**	**Cronbach’s α**	Mean±SD *
Total work environment	26	26~156	0.957	3.75±1.010
Professional development	5	5~30	0.870	3.79±1.021
Support and care	4	4~24	0.890	3.70±0.984
Nurse-physician relationship	4	4~24	0.907	3.81±0.932
Recognition of value	3	3~18	0.882	4.13±0.923
Clinical autonomy	4	4~24	0.860	3.88±0.972
Salary and welfare	3	3~18	0.836	3.43±1.054
Staffing adequacy	3	3~18	0.900	3.40±1.016
Total CSS ^†^	11	11~55	0.916	3.42±0.765
CS ^‡^	5	5~25	0.924	3.58±0.721
PWOC ^§^	3	3~15	0.921	3.36±0.787
PEOC ^||^	3	3~15	0.904	3.21±0.751

* SD = Standard Deviation

† CCS = The Chinese Career-Success Scale

‡ CS = Career Satisfaction

§ PWOC = Perceived Within-Organization Competitiveness

|| PEOC = Perceived External Organization Competitiveness

The univariate analysis identified 12 factors that affected ART nurses’ career success: gender, marital status, presence/absence of children, educational level, average monthly income, job satisfaction, income satisfaction, presiding over or joining in scientific research projects, academic post, published a paper, undertaken teaching work, and participation in academic conferences (p < 0.05, Table 1). 

Pearson’s r-tests were conducted to examine the correlations between work environment, ART specialized nursing competence, and career success ( Table 4). There was a significant and strong positive correlation between work environment (r = 0.742, p < 0.01), ART specialized nursing competence (r = 0.540, p < 0.01) and career success, and all dimensions of the variables showed significant correlations (r = 0.237 to 0.713, p < 0.01). 

**Table 4 -:** Pearson correlations between career success and work environment in assisted reproductive technology nurses (n= 555). Mainland China, 2019

**Variables**	CCS *	**CS** ^†^	**PWOC** ^‡^	**PEOC** ^§^
**Work Environment**	0.742 ^||^	0.700 ^||^	0.713 ^||^	0.356 ^||^
Professional development	0.614 ^||^	0.581 ^||^	0.621 ^||^	0.259 ^||^
Support and care	0.623 ^||^	0.551 ^||^	0.617 ^||^	0.334 ^||^
Nurse-physician Relations	0.587 ^||^	0.554 ^||^	0.556 ^||^	0.290 ^||^
Recognition of value	0.541 ^||^	0.564 ^||^	0.458 ^||^	0.243 ^||^
Clinical autonomy	0.566 ^||^	0.539 ^||^	0.488 ^||^	0.323 ^||^
Salary and welfare	0.643 ^||^	0.612 ^||^	0.641 ^||^	0.276 ^||^
Staffing adequacy	0.599 ^||^	0.545 ^||^	0.681 ^||^	0.274 ^||^

* CCS = The Chinese Career-Success Scale

† CS = Career Satisfaction

‡ PWOC = Perceived Within-Organization Competitiveness

§ PEOC = Perceived External Organization Competitiveness

|| p<0.001

The 12 significant variables from the univariate analyses, seven subscales of the work environment, and six variables of the specialised nursing competence were entered into the multiple stepwise regression model of career success. Five variables were identified in the model: attending academic conferences, psychological care, professional development, support and care, and salary and welfare ( Table 5) (adjusted *R* ^2^ = 0.605, *F* = 34.885, *p* < 0.001). 

**Table 5 -:** Multiple regression analysis of career success with associated factors in assisted reproductive technology nurses (n= 555). Mainland China, 2019

**Variables**	**Nonstandardized** **β Coefficient**	SE *	**Standardized** **β Coefficient**	**95% Confidence** **Interval**	**p**
Constant	8.000	2.299	-	[3.483, 12.517]	0.001
Attending academic conference	0.781	0.376	0.063	[0.043, 1.519]	0.038
Psychological care	0.745	0.337	0.090	[0.084, 1.406]	0.027
Professional development	0.196	0.062	0.131	[0.073, 0.318]	0.002
Support and care	0.291	0.080	0.163	[0.134, 0.449]	0.001
Salary and welfare	0.610	0.102	0.274	[0.410,0.809]	0.001
F	34.885				
R2	0.622				
Adjusted R ^2^	0.605				

* SE = Standard error

## Discussion

This study investigated Chinese ART nurses’ level of career success and their work environment and demonstrated that a positive correlation exists between them. In addition, this study identified five variables that influence career success.

Compared with previous studies, the ART nurses’ career success was at moderate to high levels (M=3.42, SD=0.77), and it was significantly higher than that of nurses in emergency departments@b17 (M=2.86,SD=0.51), and nurses with a master’s or doctoral degree ^( 10)^ (mean=3.18, standard deviation=0.72) in China. The results indicate that ART nurses have better career success. The subscale findings were similar to those for nurses with a master’s or doctoral degree ^( 10)^ , and career satisfaction was the highest ranked, which further confirms previous researcher’s suggestion that staff members in infertility settings have a fairly high degree of job satisfaction ^( 17)^ . 

The high career satisfaction score may be due to the following reasons. First, the ART nurses in our study were relatively young, and most had a bachelor’s degree. Second, they generally had multiple responsibilities, including providing nursing care, auxiliary diagnosis, treatment plan management, consultation, and patient education, which were linked at all levels with the patients, clinicians, and related disciplines. This nursing practice may significantly contribute to patient care and promote self-development. Third, the job satisfaction of employees working fixed nights is reduced ^( 18)^ . A review of job satisfaction among nurses working in hospitals found that nurses working in shifts had a lower level of job satisfaction than those working only daytime shifts, and that nurses without weekly night shifts had a higher level of job satisfaction than those with two or more night shifts ^( 19)^ . However, in our study, more than three-quarters of the nurses did not have night shifts, which may enhance their work and career satisfaction. Fourth, the PEOC subscale had the lowest score, followed by the PWOC subscale. These results may relate to the lack of unified management standards for Chinese fertility centres, which makes nurses less competitive among different infertility settings. Of the ART nurses in our study, 42.5% had worked in infertility settings for less than five years; therefore, they are more eager to develop a career within their own hospital platform, which limits the flow of quality care resources. To promote continuing professional development, organizations need to recognize nurses’ personal goals and unique strategies and provide available and accessible resources ^( 12)^ . 

In the present study, the ART nurses reported a moderate level of satisfaction with the work environment, which is lower than that for registered nurses in China ^( 20)^ . Our findings showed that recognition of value was scored highest by the respondents, which may be because fertility nursing is a specialised practice area where nurses are at the forefront of emerging care that involves a wide range of highly specialised skills ^( 14)^ . However, staffing adequacy was scored the lowest, which is consistent with the findings of other studies ^( 21)^ . As infertility rates increase, so does the number of individuals seeking infertility treatment from healthcare professionals ^( 22)^ . A systematic review showed that an increased nurse-to-patient ratio increased the burnout levels of nurses ^( 23)^ , thus, inadequate nurse staffing becomes a more serious problem. Additionally, ART nurses have multiple roles and are burdened with heavy workloads, and patient- centred care is an important criterion when patients are selecting fertility clinics ^( 24)^ , which can make the nurses’ workloads heavier. We found that the work environment was positively and strongly related to career success. In addition, our results showed that the seven subscales of the nursing work environment affected the nurses’ career success. We thus conclude that a better work environment contributes to ART nurses’ greater career success. 

We found that attending academic conferences was positively related to career success. Attending conferences is an excellent way to obtain an up-to-date perspective on what is happening in the field, and is an important part of an academic career ^( 25)^ . One reason is that attendees are required to present key findings and ideas about how these can be used to improve practice in their workplace after the conference ^( 26)^ . Another reason is that, for Chinese nurses, achieving continuing education credits by attending academic conferences is an indispensable part of promotion. Thus, we suggest that fertility centre managers provide opportunities for nurses to attend academic conferences. 

In the multiple regression analysis, we found that psychological care was positively associated with career success. It is widely recognised that psychosocial care is important in infertility care, because most patients experience emotional distress during treatment due to the infertility itself, high costs, invasive medical procedures, and unpredictable outcomes ^( 27)^ . Psychological intervention has a significant effect on reducing negative emotions ^( 27)^ , which may strengthen the nurses’ occupational achievement. The European Society of Human Reproduction and Embryology developed a guide that offers evidence- based best practice advice to all fertility clinic staff on how to incorporate psychosocial care into routine infertility care. According to the guide, ART nurses should inform themselves of the patient’s specific needs at different treatment stages and tailor psychosocial care accordingly ^( 28)^ . 

Consistent with recent literature ^( 10)^ , our study identified the work environment as a positive aspect of career success. The subscales of professional development, support and care, and salary and welfare were associated with career success. Professional development, and salary and welfare, appear to reflect organisational traits at the hospital level ^( 15)^ . Promoting optimal assisted reproduction outcomes and emphasising innovation and evidence-based practice are important challenges for ART nurses. Strong career development support, like the defined registered nurse career ladder, and organisational investment in nurses’ knowledge and skill development, may minimise turnover by displaying their organisational commitment to employees ^( 29)^ . 

Salary level is considered to be an objective measure of career success, and research with Italian ^( 30)^ , Iranian ^( 31)^ and Turkish ^( 32)^ nurses found that the higher the income, the higher the job satisfaction and career success. Welfare refers to the Chinese social insurance system, which includes endowment insurance, medical insurance, unemployment insurance, maternity insurance, occupational injury insurance, and housing funds. Salary and welfare play an important role in the nursing work environment ^( 33)^ . Support and care that is focused on social relationships typically occurs at the unit level, and leadership and management highlights the supervisors’ support and assistance ^( 15)^ . A systematic review showed that leadership affects the nursing workforce and work environment ^( 34)^ . Organisations and individuals should encourage and develop transformational and relational leadership to enhance nurses’ satisfaction, recruitment, retention, and healthy work environments. 

This study has several limitations. First, it failed to collect some basic information from the 53 fertility centers that may potentially affect career success (e.g., annual outpatient service, annual IVF cycle number, annual live births, and number of registered nurses). Exploring these factors that lead to career success in future studies would provide policymakers with a clearer idea. Second, this study is limited by its cross-sectional design, and causal relationships cannot be established. Thus, a longitudinal design is needed in future studies to find definitive cause and effect over time.

## Conclusion

This multicentre cross-sectional study demonstrates that attending academic conferences, psychological care, professional development, support and care, and salary and welfare are significantly associated with career success for Chinese ART nurses. Given that ART nurses play a very important role in providing infertility care during ART treatment, we suggest that the administrators of infertility settings pay attention to ART nurses’ career success, provide more opportunities for them to attend academic conferences, and strengthen their psychological care skills. Moreover, we suggest that fertility centres train administrators in management and leadership ability, strengthen their care and support for nurses’ professional development, and create a good working environment, to promote ART nurses’ career success. Further research is needed to retain more ART nurses.

## References

[ref-b1] Zegers-Hochschild F., Adamson G. D., Dyer S., Racowsky C., Mouzon J., Sokol R. (2017). The international glossary on infertility and fertility care, 2017. Hum Reprod.

[ref-b2] Vander Borght M, Wyns C (2018). Fertility and infertility: Definition and epidemiology. Clin Biochem.

[ref-b3] Peters K. (2003). In pursuit of motherhood: The IVF experience. Contemp Nurse.

[ref-b4] Gameiro S., Boivin J., Peronace L., Verhaak C. M. (2012). Why do patients discontinue fertility treatment? A systematic review of reasons and predictors of discontinuation in fertility treatment. Human Reprod Update.

[ref-b5] Boivin J., Takefman J., Braverman A. (2011). The fertility quality of life (FertiQoL) tool: Development and general psychometric properties. Hum Reprod.

[ref-b6] Liu W., Kang X., Fang Y., Fang M. (2018). Analysis of current status and influencing factors of occupational burnout among 116 nurses in reproductive department. J Nurs.

[ref-b7] Queiroz A. B. A., Mohamed R. P. S., Moura M. A. V., Souza I. E. O., Carvalho M. C. M. P., Vieira B. D. G. (2020). Nursing work in assisted human reproduction: Between technology and humanization. Rev Bras Enferm.

[ref-b8] Li Z. K., You L. M., Lin H. S., Chan S. W. (2014). The career success scale in nursing: Psychometric evidence to support the Chinese version. J Adv Nurs.

[ref-b9] Wu C., Zhang L. Y., Zhang X. Y., Du Y. L., He S. Z., Yu L. R. (2022). Factors influencing career success of clinical nurses in northwestern China based on Kaleidoscope Career Model: Structural equation model. J Nurs Manag.

[ref-b10] Wang Y., Zhang L., Tian S., Wu J., Lu J., Wang F. (2019). The relationship between work environment and career success among nurses with a master’s or doctoral degree: A national cross-sectional study. Int J Nurs Pract.

[ref-b11] Dan X., Xu S., Liu J., Hou R., Liu Y., Ma H. (2018). Relationships among structural empowerment, innovative behaviour, self-efficacy, and career success in nursing field in mainland China. Int J Nurs Pract.

[ref-b12] Hakvoort L., Dikken J., Cramer-Kruit J., Nieuwenhuyzen K. M., Schaaf M., Schuurmans M. (2022). Factors that influence continuing professional development over a nursing career: A scoping review. Nurse Educ Pract.

[ref-b13] Zamanzadeh V., Valizadeh L., Praskova A., Ghahramanian A., Rassouli M., Asghari E. (2019). Reaching for the stars: Iranian nurses’ perceptions of career success. Int Nurs Rev.

[ref-b14] Nantsupawat A., Kunaviktikul W., Nantsupawat R., Wichaikhum O. A., Thienthong H., Poghosyan L. (2017). Effects of nurse work environment on job dissatisfaction, burnout, intention to leave. Int Nurs Rev.

[ref-b15] Mitchell A., Mittelstaedt M. E., Wagner C. (2005). A survey of nurses who practice in infertility settings. J Obstet Gynecol Neonatal Nurs.

[ref-b16] Shao J., Tang L., Ye Z. (2017). Measuring the nursing work environment in mainland china: Further development of the chinese nursing work environment scale. Nurs Res.

[ref-b17] Han F., Wang Z. (2017). The relationship between the working environment and the sense of career success of nurses in the emergency department. Chin Nurs Manage.

[ref-b18] Gerson S. C., Kemp D. E., Balser D. P., Masler S. N., Hart B., Bubka A. (2004). Infertility practice management. I. Leadership and management style: Results from the 2002 survey of 374 Society for Assisted Reproductive Technology member centers. Fertil Steril.

[ref-b19] Dall’Ora C., Ball J., Recio-Saucedo A., Griffiths P. (2016). Characteristics of shift work and their impact on employee performance and wellbeing: A literature review. Int J Nurs Stud.

[ref-b20] Lu H., Zhao Y., While A. (2019). Job satisfaction among hospital nurses: A literature review. Int J Nurs Stud.

[ref-b21] Shao J., Tang L., Wang X., Qiu R., Zhang Y., Jia Y. (2018). Nursing work environment, value congruence and their relationships with nurses’ work outcomes. J Nurs Manage.

[ref-b22] Nantsupawat A., Kunaviktikul W., Nantsupawat R., Wichaikhum O. A., Thienthong H., Poghosyan L. (2017). Effects of nurse work environment on job dissatisfaction, burnout, intention to leave. Int Nurs Rev.

[ref-b23] Stevenson E. L., Hershberger P. E., Bergh P. A. (2016). Evidence-based care for couples with infertility. J Obstet Gynecol Neonatal Nurs.

[ref-b24] Shin S., Park J. H., Bae S. H. (2018). Nurse staffing and nurse outcomes: A systematic review and meta-analysis. Nurs Outlook.

[ref-b25] IW Empel, EA Dancet, XH Koolman, WL Nelen, EA Stolk, W Sermeus (2011). Physicians underestimate the importance of patient-centredness to patients: a discrete choice experiment in fertility care. Hum Reprod.

[ref-b26] Hershberger P. E., Minton M., Voss J. G., McCarthy A. M., Murrock C. J., Topp R. (2019). Midcareer faculty needs identified by the midwest nursing research society midcareer scholars task force. West J Nurs Res.

[ref-b27] Stone T. E., Rossiter R. (2014). Making the most of conference attendance. Nurs Health Sci.

[ref-b28] Frederiksen Y., Farver-Vestergaard I., Skovgard N. G., Ingerslev H. J., Zachariae R. (2015). Efficacy of psychosocial interventions for psychological and pregnancy outcomes in infertile women and men: A systematic review and meta-analysis. BMJ Open.

[ref-b29] Gameiro S., Boivin J., Dancet E., Klerk C., Emery M., Lewis-Jones C. (2015). ESHRE guideline: Routine psychosocial care in infertility and medically assisted reproduction-a guide for fertility staff. Hum Reprod.

[ref-b30] Lindley L. C., Cozad M. J. (2017). Nurse knowledge, work environment, and turnover in highly specialized pediatric end-of-life care. Am J Hosp Pall Care.

[ref-b31] Sansoni J., Caro W., Marucci A. R., Sorrentino M., Mayner L., Nurses’ Lancia L. (2016). Job satisfaction: An Italian study. Ann Ig.

[ref-b32] Atefi N., Lim Abdullah K., Wong L. P., Mazlom R. (2015). Factors influencing job satisfaction among registered nurses: a questionnaire survey in Mashhad, Iran. J Nurs Manage.

[ref-b33] Sönmez B., Gül D., İspir Demir Ö., Emiralioğlu R., Erkmen T., Yıldırım A. (2021). Antecedents and outcomes of nurses’ subjective career success: A path analysis. J Nurs Scholarsh.

[ref-b34] Lin C. F., Lu M. S., Huang H. Y. (2016). The psychometric properties and the development of the indicators of quality nursing work environments in taiwan. J Nurs Res.

[ref-b35] Cummings G. G., Tate K., Lee S., Wong C. A., Paananen T., Micaroni S. P. M. (2018). Leadership styles and outcome patterns for the nursing workforce and work environment: A systematic review. Int J Nurs Stud.

